# A novel locus for mycelial aggregation forms a gateway to improved *Streptomyces* cell factories

**DOI:** 10.1186/s12934-015-0224-6

**Published:** 2015-04-01

**Authors:** Dino van Dissel, Dennis Claessen, Martin Roth, Gilles P van Wezel

**Affiliations:** Molecular Biotechnology, Institute of Biology, Leiden University, PO Box 9505, 2300RA Leiden, The Netherlands; Bio Pilot Plant, Leibniz Institute for Natural Product Research and Infection Biology, Hans Knöll Institute, Adolf-Reichwein-Str. 23, 07745 Jena, Germany

**Keywords:** Reverse engineering, Morphology, Genome sequencing, Pellet, Actinomycete, Antibiotic

## Abstract

**Background:**

Streptomycetes produce a plethora of natural products including antibiotics and anticancer drugs, as well as many industrial enzymes. Their mycelial life style is a major bottleneck for industrial exploitation and over decades strain improvement programs have selected production strains with better growth properties. Uncovering the nature of the underlying mutations should allow the ready transfer of desirable traits to other production hosts.

**Results:**

Here we report that the *mat* gene cluster, which was identified through reverse engineering of a non-pelleting mutant selected in a chemostat, is key to pellet formation of *Streptomyces lividans*. Deletion of *matA* or *matB*, which encode putative polysaccharide synthases, effects mycelial metamorphosis, with very small and open mycelia. Growth rate and productivity of the *matAB* null mutant were increased by over 60% as compared to the wild-type strain.

**Conclusion:**

Here, we present a way to counteract pellet formation by streptomycetes, which is one of the major bottlenecks in their industrial application. The *mat* locus is an ideal target for rational strain design approaches aimed at improving streptomycetes as industrial production hosts.

**Electronic supplementary material:**

The online version of this article (doi:10.1186/s12934-015-0224-6) contains supplementary material, which is available to authorized users.

## Background

Members of the genus *Streptomyces* are of great importance for biotechnology due to their ability to produce a large array of natural products, including antibiotics, anticancer agents and immunosuppressants [1], as well as a plethora of industrially relevant enzymes, such as cellulases, amylases and proteases [2]. As surface-grown cultures, streptomycetes exhibit a complex multicellular life cycle [3]. This starts with a single spore that germinates to form vegetative hyphae, which then grow out following a process of hyphal growth and branching to produce a branched vegetative mycelium [4]. Nutrient scarcity or other stresses induce the developmental program, whereby aerial hyphae differentiate into long chains of spores following a complex cell division event whereby ladders of septa are produced within a short time span [5,6]. In a submerged environment streptomycetes grow as mycelial networks, typically forming large pellets or clumps. From the industrial perspective, growth as pellets is unattractive, in particular because of mass-transfer problems, slow growth and culture heterogeneity (reviewed in [7]). Pellets restrict the efficient transfer of nutrients and gasses to the centre, which lowers the maximal obtainable product yield [8]. The growth rate is also limited by the rate at which new pellets can be formed, which requires fragments of viable mycelia to be released from the pellet. Fragmentation is highly depended on the shear forces present in the environment [9,10]. Because high shear can cause cell damage it needs to be balanced for efficient growth and mass transfer and therefore production [11].

Industrial strain optimization programs often make use of black-box approaches to select for desirable traits, typically using mutagens or protoplast fusion [12]. As an example, penicillin yield has been improved at least three orders of magnitude since the isolation of the original *Penicillium chrysogenum* strain. In the improvement program the growth characteristics were also improved upon, which contributed both to the production titres and the fermentability of the strain [13]. Production of clavulanic acid by *Streptomyces clavuligerus* underwent a similar improvement program [14]. Classical strain improvement, however, is also slow and labour-intensive, and typically associated with a large number of mutations that may later slow down further improvement. Rational strain design requires understanding of the system, but changes can be made fast. Also, directed mutagenesis should result in fewer additional mutations and the changes can be transferred from one strain to another [15]. The latter is of particular importance for *Streptomyces*, where secondary metabolites cannot easily be moved to generally preferred production host such as *E. coli* or *B. subtilis*.

Relatively little is known of the genetic factors that control pellet morphology in submerged cultures of *Streptomyces*. Growth rate and enzyme production by *S. lividans* are improved significantly by inducing mycelial fragmentation via the increased expression of the cell division activator protein SsgA [16]. SsgA belongs to the family of SsgA-like proteins that occur exclusively in actinomycetes [17], and SsgA not only effects a significant increase in the number of septa, but also has a major impact on the overall hyphal morphology [18,19]. Several other proteins have been identified that affect morphology in submerged culture. These include HyaS, which is involved in cell-wall fusion [20] and CslA, a cellulose synthase-like protein that is required for pellet formation [21].

To search for novel genes that may be applied for growth improvement, reverse engineering of randomly obtained mutants is a logical approach that has become feasible in the genomics era [15,22]. This allows identification of the mutations that have been sustained during the development of industrial production strains. Together with metabolic engineering and synthetic biology approaches, this accelerates the development of microbial factories, exemplified by industrial isobutanol production by *Escherichia coli* [23], ethanol production by yeast [24] and lysine or glutamate production by the industrial actinomycete *Corynebacterium glutamicum* [25,26].

A regime to improve growth of *Streptomyces lividans* 66 by selection in a chemostat for over 100 generations resulted in a stable derivative with a non-pelleting phenotype (PM02), and an intermediate mutant forming loose pellets (PM01) [27]. PM02 was used to study the segregational stability of plasmids in continuous culture with different growth limiting substrates [27,28]. In this work, we identified the mutations accumulated during the generation of *S. lividans* 66-PM01 and 66-PM02. The mutation responsible for the non-pelleting phenotype was subsequently identified in a gene for a membrane protein that is co-transcribed with a gene for a bifunctional polysaccharide deacetylase/synthase. Both of these genes, designated *matA* and *matB*, respectively, were shown to be required for pellet formation. Thus, reverse engineering elucidated a novel molecular determinant underlying pellet morphogenesis.

## Results

### Derivatives of *S. lividans* 66 with improved growth characteristics

**Figure 1:**
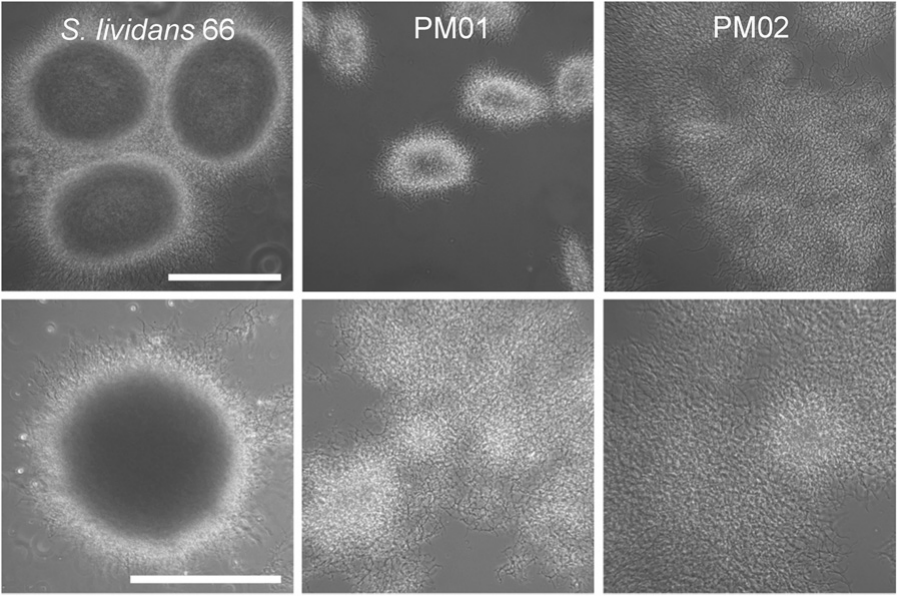
**Morphology of** ***S. lividans*** **66 and its derivatives PM01 and PM02 in submerged culture.** PM01 was obtained after 70 generations in a nitrogen-limited chemostat at a dilution rate of 0.1 h^-1^. PM02 was obtained after evolving PM01 for another 30 generations in a phosphate-limited chemostat at 36°C and a dilution rate of 0.1 h^-1^. Top row: growth of strains in a baffled shake flask for 30 h on TSBS. Bottom row: cultivation of strains in a 900 ml bioreactor in TSBS media after 24 h of growth. Scale bar, 200 μm.

**Figure 2:**
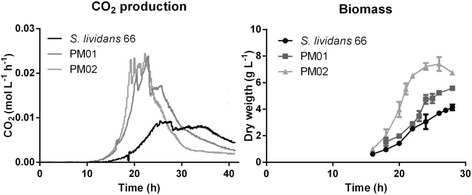
**Growth of** ***S. lividans*** **66 and its derivatives PM01 and PM02 in a bioreactor.** 1.3 liter reactors were inoculated with 10^6^ spores/mL in TSBS medium. CO_2_ levels were measured by an off-gas analyser and biomass accumulation was measured as dry weight from 10 mL freeze-dried broth that was collected and washed on a glass fibre filter. The data shown are the average of three separate fermentations. Error bars in the biomass graphs represent the standard error of the mean; the CO_2_ data had a deviation of less than 5%.

Many *Streptomyces* species produce large mycelial clumps when grown in liquid media, which is a disadvantage for industrial application as it is associated with slow growth and poor nutrient utilization. In an attempt to obtain faster growth with less dense pellets, a non-pelleting derivative of *S. lividans* 66 (also known as *S. lividans* 1326) was obtained previously through growth of some 100 generations in continuous culture at a medium dilution rate, called PM02, while a strain with an intermediate phenotype was obtained after 70 generations, called PM01, which grows as small loosely packed mycelial pellets (Figure 1). While the parent grew as large pellets with an average diameter of around 250 μm, the pellets of PM01 were much smaller, averaging around 150 μm. PM02 did not form any mycelial pellets, except that on increasing biomass densities patches of dispersed mycelia entangled into open pellet-like structures. To quantitatively describe the growth characteristics, the strains were compared in a bioreactor with TSBS as the growth medium. On TSBS medium PM02 had a maximal growth rate of around 0.41 ± 0.09 h^−1^ (doubling time of 1.7 h), which was significantly faster than the parental strain that had a maximal growth rate of around 0.25 ± 0.02 h^−1^ (doubling time of 2.7 h) under the chosen conditions. The benefits of a dispersed morphology, a higher average growth rate and shorter batch duration, are clearly present (Figure 2). In contrast to shake flask cultivation, under the conditions in the bioreactor PM01 also grew dispersed, resulting in a growth rate of 0.36 ± 0.06 h^−1^ (1.9 h doubling time). The difference between growth in shake flasks and in a bioreactor in terms of the morphology is likely explained by the differences in shear and aeration.

### Genome analysis of *S. lividans* PM01 and PM02

To investigate the genetic basis for the changes in phenotypes in derivatives PM01 and PM02 relative to the parental strain, we analysed the single nucleotide polymorphisms (SNPs). For this, whole genome sequencing was performed on the parental strain and on PM01 and PM02 using illumina paired-end sequencing and the sequences were then compared to the draft genome sequence of *S. lividans* 66 [29]. The genome sequence of the parental strain was used to filter out all nt changes relative to the published draft genome. After this filtering step, we identified in total 19 SNPs, 17 between PM01 and the parent, and two more between PM02 and the parent. These mutations resulted in 10 aa substitutions and four frameshifts in putative proteins of PM01 relative to the parental strain *S. lividans* 66 and an additional two aa changes in PM02 relative to PM01 (Table 1). Additionally, the *bldA* gene for the tRNA-Leu that recognises the rare codon UUA in the mRNA was also mutated in PM01 and PM02. This mutation probably explains the developmental block in both strains, as *bldA* mutants fail to erect an aerial mycelium [30,31].

**Table 1:** **SNPs identified in mutants PM01 and PM02 as compared to the wild-type reference strain** ***S. lividans*** **66**

**Genomic variants detected in PM01**						
***Postion***	***Refence***	***Variant***	***codonChange***	***Sli GN***	***Sco GN***	***Description***
1188051	C	T	Gly344Asp	SLI_1181	SCO0948	Alpha-mannosidase
2924224	G	A	Ala104Thr	SLI_2849*	SCO2513	Putative DNA-binding protein
3429004	G	-	Leu165fs	SLI_3306a*	SCO2963	Putative membrane protein
3586487	C	T	C16T	SLI_10025	*bldA*	tRNA-Leu
3717404	C	T	Val70lle	SLI_3556	SCO3202	RNA polymerase sigma factor
4336711	-	C	Gly121fs	SLI_4122	SCO3868	Uncharacterized protein
4499627	C	G	Ala83Pro	SLI_4277	SCO4043	Uncharacterized protein
4568325	G	A	Pro119leu	SLI_4341	SCO4111	tRNA (guanine-N(7)-)-methyltransferase
4967084	A	-	Leu130fs	SLI_4756	SCO4477	Transcriptional regulator, MerR family
5479836	A	CC	Ala67fs	SLI_5273*	SCO4998	DNA-binding protein
5700075	-	A	Val30Asp	SLI_5478	SCO5200	Putative membrane
6362450	A	G	Thr271Ala	SLI_6089*	SCO5821	Putative serine proteinase
6513051	C	T	Pro142Leu	SLI_6232*	SCO5952	Uncharacterized protein
6750912	G	C	Ala27Pro	SLI_6469*	SCO6076	Putative dipeptidase
7560900	A	C	Thr484Ala	SLI_7172	SCO6968	Long-chain-fatty acid--CoA ligase
**Additional genomic variants detected in PM02**						
3537971	G	A	VAl496lle	SLI_3391*	SCO3043	Cell envelope-associated transcriptional attenuator LytR-CpsA-Psr
6420301	G	C	Ala652Pro	SLI_6143*	SCO5871	Osmosensitive K+ channel histidine kinase KdpD

The nt position refers to the published genome sequence of *S. lividans* [29]. Only non-silent mutations inside CDSs are shown. Genes followed by an asterisk were selected for targeted gene disruption. Database numbers for *S. lividans* (Sli GN) and *S. coelicolor* (Sco GN) are given, based on the nomenclature of the StrepDB database (http://strepdb.streptomyces.org.uk).

### Analysis of mutations that may relate to the PM02 phenotype

To identify the mutations that gave rise to the non-pelleting phenotype of PM01 and PM02, we used directed mutagenesis followed by morphological characterization of the mutant derivatives in comparison to PM01 and PM02. In total seven genes were initially prioritized for genomic disruption (highlighted in grey in Table 1). Of these, five were found in PM01: SLI_2849, SLI_5273 and SLI_6232 for putative DNA binding proteins, SLI_6089 for a serine protease, which is located next to the principal sigma factor gene *hrdB* and SLI_6469 for a dipeptidase. The two additional mutations found only in PM02 were in SLI_3391, which encodes a LytR-type transcriptional attenuator associated with cell-wall remodelling and biofilm formation [32-34] and in SLI_6143 for an osmosensitive potassium channel histidine kinase (KdpD). A gene-disruption library is available for the majority of the *S. coelicolor* genes*,* whereby genes have been mutated on cosmids using transposon mutagenesis, which facilitates a rapid first assessment of gene function [35]. These gene-disruption cosmids were used to create mutants in the seven genes mentioned above in both *S. lividans* 66 and *S. coelicolor* M145*.* For exact genomic position of the transposon insertion we refer to Additional file 1: Table S2. Disruption of SLI_3391 (SCO3043), resulted in a white (non-sporulating) phenotype on solid media and pellets with a slightly more open perimeter in liquid-grown cultures (Additional file 1: Figure S1). Likewise, mutants deleted for either SLI_6143 (SCO5871) or SLI_6089 (SCO5821), had a phenotype with a slightly decreased pellet density, but this did not compare to the drastic morphological changes seen in the PM01 and PM02 lineages.

Since none of these mutations could explain the non-pelleting phenotype of PM01 and PM02, we scrutinized the list of SNPs further. This identified a conspicuous mutation 216 bp upstream of SLI_3306, which encodes a bi-functional transferase/deacetylase. The predicted protein has an N-terminal NodB-like polysaccharide deacetylase domain, and a C-terminal PgaC-like glycosyltransferase type 2 domain. Comparison of the genomic region to that of the close relative *S. coelicolor* revealed that the SNP upstream of SLI_3306 corresponds to a position *inside* the annotated gene SCO2963, which encodes a putative membrane protein that is well conserved in streptomycetes. This gene is translationally coupled with SCO2962. Resequencing of the region between SLI_3306 and the downstream SLI_3307 revealed a mistake in the *S. lividans* 66 genome sequence, and in fact the SNP lies inside an orthologue of SCO2963 with very high similarity between the predicted gene products (99% aa identity). This gene was designated SLI_3306a. The deletion of nt position 3429004 in SLI_3306a found in strain PM01 results in a frame shift which introduces a premature stop codon at amino acid residue 176, most likely rendering the SLI_3306a protein non-functional. Since SLI_3306a and SLI_3306 have overlapping stop and start codons and are therefore most likely translationally coupled, the premature termination of SLI_3306a may have major consequences for the translational efficiency of SLI_3306 and other putative co-translated genes [36].

### Deletion of SLI_3306/3306a or SCO2962 causes a non-pelleting phenotype

**Figure 3:**
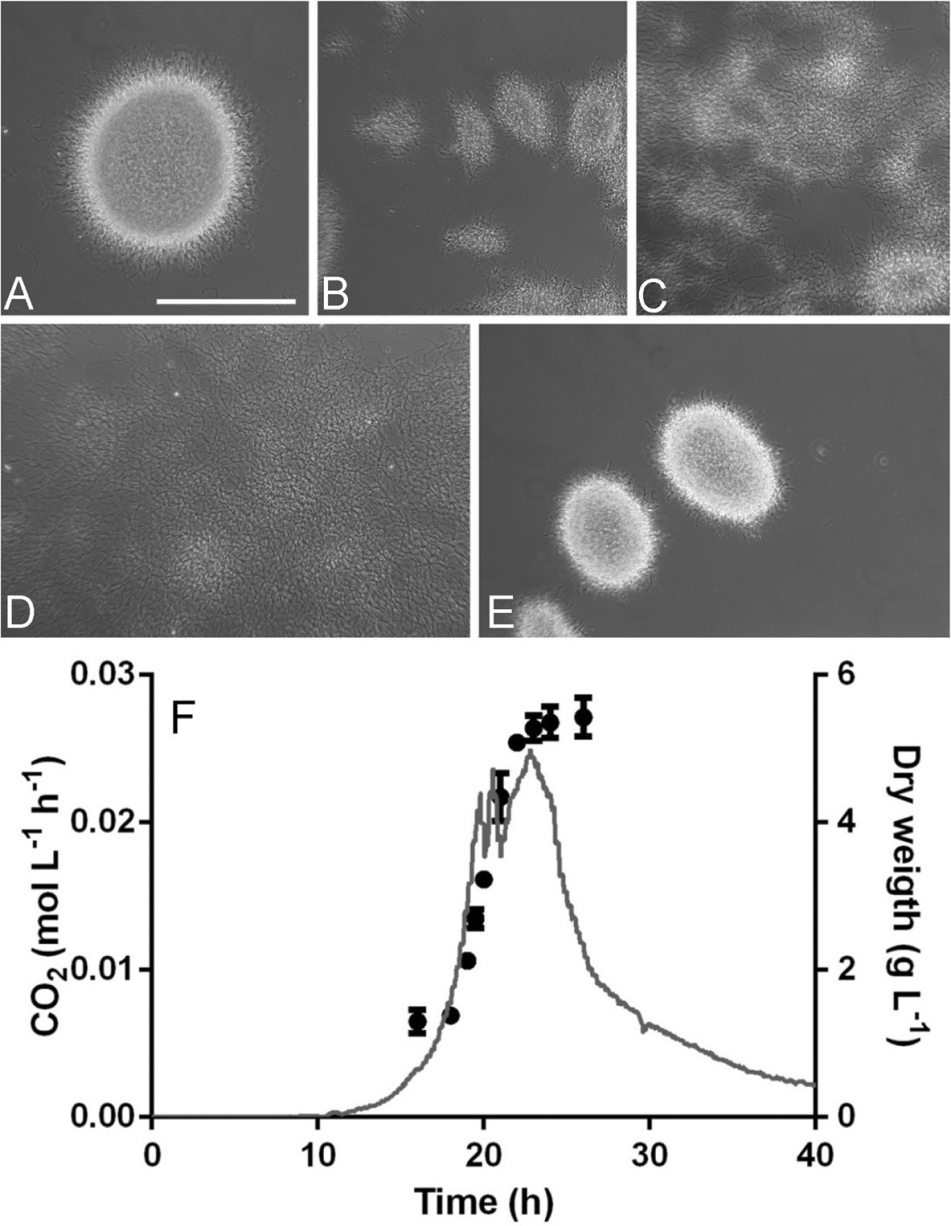
**Liquid-culture morphology of the** ***S. lividans mat*** **mutants. A-E** represent wide field images of strains grown in shake flasks in TSBS media. **(A)** wild type *S. lividans* 66; **(B)** GAD02 (*S. lividans* ∆*matA*); **(C)** GAD03 (*S. coelicolor* ∆*matB*); **(D)** GAD05 (*S. lividans* ∆*matAB*); **(E)** *S. lividans* PM02 complemented with plasmid pMAT03 expressing wild-type *matA*. Pictures were taken at 24 h of growth representing late exponential growth. Bar, 200 μm. **(F)** growth profile of GAD05, indicated by the CO_2_ production (grey line) and the biomass concentration (black dots). The strain was grown in a 1.3 L benchtop bioreactor in TSBS medium. Inoculation density was 10^6^ spores/mL. The strain had a growth rate of 0.39 ± 0.02 h^−1^, which was calculated from the measured biomass from three separate fermentation experiments.

To investigate the role of SLI_3306 and SLI_3306a in mycelial morphogenesis, both genes were simultaneously replaced with the apramycin resistance cassette *aacC4* by homologous recombination. The *aacC4* gene was flanked by *loxP* sites, allowing the subsequent removal by expression of the Cre recombinase, resulting in a clean deletion of the genes and leaving only the start region of SLI_3306a and the stop region of SLI_3306 (see Materials and Methods section for details). The removal of the region yielded the strains GAD02 (∆SLI_3306a) and GAD05 (∆SLI_3306a + ∆SLI_3306) (Figure 3). GAD02 showed an altered morphology with small pellets, while the removal of both *mat* genes yielded a highly dispersed phenotype. Considering their apparent involvement in mycelial aggregation, SLI_3306a and SLI_3306 were renamed *matA* and *matB* (for **m**ycelial **a**ggrega**t**ion).

Analysis of a number of streptomycetes showed that the *mat* genes and flanking regions are conserved in around two-thirds of all *Streptomyces* genomes (not shown). Besides *S. coelicolor* and *S. lividans,* these include for example *S. albus, S. griseus, S. hygroscopicus* and *S. avermitilis*. The *mat* cluster was not found in *S. venezuelae* (which hypersporulates in submerged culture) or in *S. clavuligerus* (which forms large mycelial mats). A more detailed phylogenetic analysis is required to establish the possible correlation, if any, between morphogenesis and the presence of the *mat* genes.

We also analysed a mutant of *S. coelicolor ∆matB* (SCO2962, the orthologue of SLI_3306). This mutant was created using the Redirect strategy [37], and had a phenotype that was highly similar to that of the *S. lividans matAB* null mutant (Figure 3), providing further evidence that the non-pelleting phenotype indeed correlates to the *mat* locus and also that the phenomenon is more widely applicable than only in *S. lividans*. The mutants were then complemented with a wild-type copy of *matA* to establish if the mutation was indeed the sole cause of the non-pelleting phenotype. For this, the entire *matA* gene and 500 bp upstream (promoter) region were amplified by PCR from the *S. lividans* genome and cloned into the integrative vector pSET152, generating plasmid pMAT04. This plasmid integrates at the ΦC31 attachment site on the genome. Introduction of pMAT04 completely restored pellet formation to mutant PM02, resulting in a wild-type morphology (Figure 3). This strongly suggests that the frameshift inside SLI_3306a is indeed the main cause for the observed metamorphosis.

The growth characteristics of the *matAB* double mutant GAD05 were tested in small-scale bioreactors to compare them with the original mutant PM01 and PM02. Growth of the mutant on TSBS showed striking similarity with the growth profile to PM02, with a completely dispersed morphology throughout the cultivation, reaching a maximal growth rate of 0.39 ± 0.005 h^−1^ (doubling time of 1.8 h; Figure 3), which is highly similar to that of PM02 (0.41 h^−1^; doubling time of 1.7 h).

### Effect of the *mat* mutation on yield

**Figure 4:**
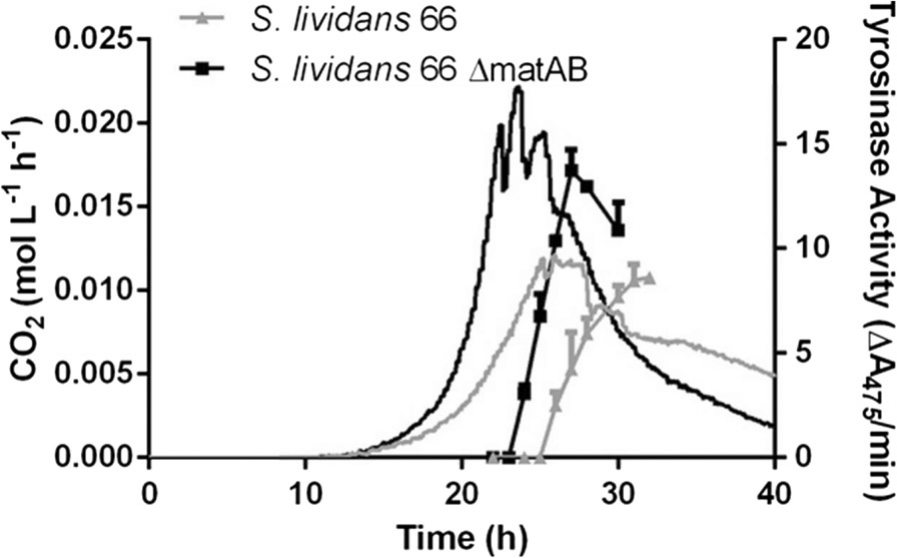
**Effect of deletion of** ***matAB*** **on tyrosinase production by** ***S. lividans*** **.** Transformants of the parental strain *S. lividans* 66 (grey curves) and its *matAB* null mutant (black curves) contained plasmid pIJ703, leading to the expression of tyrosinase (lines with blocks) from *S. antibioticus*. Strains were grown in 1.3 L bench-top bioreactors with a 900 ml working volume on TSBS medium with 25 μM CuCl_2_. CO_2_ production (continues lines) was calculated by measuring the concentration in the off gas and tyrosinase activity was measured spectrophotometrically. The CO_2_ production and tyrosinase activity is the average of three separate experiments. Error bars in the biomass graphs represent the standard error of the mean and the CO_2_ had a deviation of less than 5%.

As an initial test to establish the effect of the non-pelleting phenotype for production in a bioreactor, we compared the ability of GAD05 with its parent *S. lividans* 66 to produce the secreted enzyme tyrosinase, which is secreted via the Twin Arginine Transport (Tat) pathway [38]. For this, plasmid pIJ703 harbouring *melC2* for tyrosinase was introduced into both strains. Tyrosinase activity can easily be measured via an enzyme assay, and is therefore a very suitable reporter protein for heterologous protein production in *Streptomyces* [16]. Growth and productivity of *S. lividans* 66 and its *matAB* double mutant GAD05 was compared in a 1.3 L bioreactor in TSBS media (Figure 4).

The morphology of mutant GAD05 positively contributed to the production capacity of *S. lividans*, with more enzyme produced in a shorter time (Figure 4). Notably, also under production conditions the specific growth rate increased by nearly 60% from 0.25 h^−1^ to 0.39 h^−1^. A tyrosinase enzyme assay based on the conversion of dihydroxy-L-phenylalanine revealed that the maximal tyrosinase activity increased by around 65%, from 8.7 ± 1.0 AU to 13.7 ± 1.4 AU. Additionally, the fermentation time for GAD05 was around 5 h shorter than for its parent *S. lividans* 66. This indicates that the non-pelleting phenotype may have strong potential for biotechnological applications.

## Discussion

The applicability of chemostats in the (directed) evolution of strains has a long-standing history [39]. Because less fit variants wash out over time there is a strong selection for increased growth rates and high substrate uptake rates. The high dilution rate used for the evolution of the *S. lividans* derivatives PM01 and PM02 30 years ago [27] resulted in dramatic morphological changes of this streptomycete that normally grows as dense clumps. Instead, the derivatives produced small and open pellets (PM01, selected after 70 cycles) or complete lack of any pellets (PM02, after 100 cycles). We reverse engineered the morphology of these mutants and identified a single point mutation that was the basis for the observed mycelial metamorphosis. The phenotype associated with this mutation was designated Mat (**m**ycelial **a**ggrega**t**ion).

It is likely that other mutations also contributed to the adaptation to the high growth rate regime. The genetic complementation of the non-pelleting phenotype of PM02 by the introduction of plasmid pMAT04 - which contains wild-type *matA* - shows that the mutation in the *mat* locus is the main source for the morphogenesis, but the difference between PM01 and PM02 remains unclear. Most likely, the mutation in SLI_3306a causes the phenotype of PM01 and PM02 has sustained a secondary mutation that further progresses the dispersed phenotype. We tried to look into the effects of other SNPs found in PM02, by disrupting SLI_3391 (SCO3043) and SLI_6143 (SCO5871) in PM01. Of these, PM01ΔSLI_3391 had an altered phenotype more similar to PM02 in submerged culture (Additional file 1: Figure S2). Perhaps SLI_3391 attenuates another adhesion system that is subordinate to the Mat system, which would allow the restoration of the pelleting phenotype when *matA* was reintroduced into PM02.

What then is the function of the Mat proteins? We propose that they may fulfil a function that is similar to that of enzymes responsible for adherence and biofilm formation in other bacteria. The various systems that are responsible for enabling biofilm formation in nature utilise different ways of adherence between cells, such as extracellular DNA (eDNA) or direct contact by either cell-wall fusion or via pili [40]. Indeed, study of the literature combined with Blast analysis suggests that all these three systems may be present in *Streptomyces* spp. [20,41]. Interestingly, the genetic configuration of the *mat* operon is similar to that of the *Ica* gene cluster for the intracellular adhesion system found in *Staphylococcus* spp. [42,43]. The *Ica* operon produces a capsular extracellular polysaccharide (EPS) consisting of β-1,6-linked N-acetylglucosamine molecules, which mediates cell-cell adhesion and is required for biofilm formation [44]. This five gene system (*icaABCDR*) encodes a chitin synthase (IcaA), a polysaccharide deacetylase (IcaB), an acyltransferase (IcaC) and a membrane protein (IcaD), which is required for production of a mature EPS [45]. These genes are under the control of IcaR which controls the expression of the *icaABCD* operon. SCO2962, a predicted bifunctional polysaccharide deacetylase and glycosyltransferase, appears to be a fusion protein of IcaA and IcaB, while SCO2961 encodes an acetyl transferase, analogous to IcaC. The IclR-family regulator SCO2964 candidates as the regulator of the *mat* genes (similar to IcaR). A membrane protein homologous to IcaD is absent.

Similar to the *ica* gene cluster, the *pgaABCD* locus in *E. coli* and the *hmsHFRS* locus in *Yersinia pestis* also produce an EPS that is important for biofilm formation [46,47]. Interestingly, in the streptomycetes *S. lividans* and *S. coelicolor* the cellulose synthase-like protein CslA produces a yet undetermined EPS that plays a major role in pellet morphology in submerged cultures, with a dispersed morphology of *cslA* null mutants [21,48]. Whether either of the *mat* genes is also involved in the production of an EPS requires further investigation. Suggestively, the String database [49] indicates functional linkage between *matB* and several cell division-related genes, namely *ftsI*, for the enzyme FtsI that is involved in peptidoglycan synthesis during cell division [50], and *mraY* which is required for synthesis of the peptidoglycan precursor Lipid I [51,52]. Also, the *matAB* locus is separated by only two genes from *ftsEX* (SCO2966-SCO2967 in *S. coelicolor*), which encode the FtsEX membrane permease and associated ATPase that are required for cell division [53]. This suggests that *matAB* may relate to the synthesis of peptidoglycan rather than EPS, in particular during cell division. This may explain the absence of a membrane component similar to IcaD in the *mat* cluster. In terms of a linkage to cell division and cell-wall synthesis, it is important to note that over-expression of the cell-division activator protein SsgA has a major effect on mycelial morphology in submerged culture, with formation of mycelial mats at lower expression and small fragments and even submerged spores at high levels of expression [19]. The possible functional relationship between MatAB, SsgA and cell-wall synthesis needs to be analysed further.

*Streptomyces lividans* is a preferred host for the industrial production of enzymes from actinomycete origin [54,55]. However, its morphology with large pellets formed in submerged culture hampers productivity in the bioreactor. Availability of bioreactor capacity is of the essence, and slow growth of actinomycetes therefore imposes a major burden on reactor time. Similarly to what we report here for the effect of the *mat* mutation, the mycelial fragmentation of *S. lividans* effected by the enhanced expression of SsgA led to enhanced productivity in batch fermentation with tyrosinase as the reporter, as well as reduced fermentation times [16]. Faster growth is potentially an important step towards biotechnological application of actinomycetes for enzyme production. The strong mycelial fragmentation effected by the enhanced expression of SsgA had major consequences for antibiotic production, with enhanced production of undecylprodigiosin but a complete block in actinorhodin production [56]. The latter most likely relates to the fact that many antibiotics are produced only when pellets of a certain size are produced [57]. Importantly, we did not observe major differences in antibiotic production between *S. coelicolor* M145 and its *mat* mutants.

Besides as sources of enzymes, streptomycetes are particularly well known for their ability to produce antibiotics, anti-cancer compounds and other important natural products. Genome sequencing has revealed that actinomycetes have the potential of producing far more natural products than originally anticipated, which has led to a revival of antibiotic discovery [58]. This also raises the question as to how we can best harness the plethora of novel gene clusters that are currently being uncovered [59,60]. One logical way forward is combining synthetic biology approaches to efficiently synthesize biosynthetic gene clusters with the development of heterologous expression hosts for efficient production [61]. Examples of the latter include derivatives of *Streptomyces avermitilis* [62] and *Streptomyces coelicolor* [63] that have been stripped of many of their native antibiotic clusters. Morphological engineering should allow the development of these heterologous hosts into more efficient and cost-effective production platforms.

The obvious question to ask is, are all streptomycetes subject to growth improvement by modulating *mat* expression or deletion? Indeed, several streptomycetes lack the *mat* genes altogether. The biotechnological relevance and applicability of introduction or deletion of the *mat* genes in industrial streptomycetes, in particular with respect to enzyme and antibiotic production, is currently being investigated in more detail. Furthermore, we seek to unravel the molecular composition of the polysaccharide that most likely causes the mycelial aggregation.

## Conclusions

Our study provides new means to obtain a more dispersed morphology during fermentation, which has a positive impact on productivity, both in terms of fermentation time and yield per volume. *S. lividans mat* null mutants, designed based on reverse engineering of a mutant arisen in a chemostat, produced significantly higher titres in a shorter time frame as compared to the parent. The fact that *S. coelicolor matB* mutants showed a similar improvement of growth suggests that this may be widely applicable strategy for the rational strain design of streptomycetes that form mycelial pellets, with the aim to accelerate fermentation time and enhance productivity.

## Methods

### Bacterial strains, culturing conditions and batch fermentations

Bacterial strains used in this work are listed in Additional file 1: Table S1. *E. coli* JM109 [64] was used as host for routine cloning, and *E. coli* ET12567 [65] to produce non-methylated DNA for introduction into *Streptomyces. E. coli* ET12567 harbouring pUZ8002 was used as host for conjugative transfer of knock-out cosmids to *Streptomyces* as described [66,67]. *Streptomyces lividans 66* and *Streptomyces coelicolor* A(3)2 M145 were obtained from the John Innes Centre strain collection. Cells of *E. coli* were grown in Luria–Bertani broth (LB) at 37°C. All *Streptomyces* media and routine *Streptomyces* techniques are described in the *Streptomyces* manual [66]. R2YE (regeneration media with yeast extract) agar plates were used for protoplast regeneration, while SFM (soy flour mannitol) agar plates were used to prepare spore suspensions and SFM supplemented with 10 mM MgCl_2_ for conjugation experiments. Phenotypic characterization was done on R2YE and SFM agar plates.

For screening purposes, strains were grown in baffled shake flasks in TSBS (tryptic soy broth with 10% sucrose) for 48 h. Small-scale batch fermentations were performed in 1.3 L BioFlow 115 bioreactors (New Brunswick), at a temperature of 30°C and at constant pH (pH 7). The initial stirrer speed was set to an average 300 rpm to promote growth and fragmentation and was automatically increased to maintain a dissolved oxygen (DO) concentration above 50%. The latter was only needed during late exponential growth and therefore stirring issues did not influence the outcome of the morphological studies. Off gas was analysed by an EX-2000 (New Brunswick) and dry weight was measured by freeze-drying filtered and washed biomass obtained from 10 mL culture broth. Cultures were inoculated with spores at a density of 10^6^ cfu/mL. Reactor experiments were performed in triplicate. For the production of tyrosinase the medium was supplemented with 25 μM CuCl_2_ and 2.5 μg/mL thiostrepton [67].

### Constructs and mutants

All constructs described in this work are listed in Additional file 1: Table S3 and oligonucleotides in Additional file 1: Table S4.

#### Constructs for the deletion of SCO2963/SCO2962 and SLI_3306a/SLI3306

In-frame deletion mutants for SCO2963/SCO2962 and SLI_3306/SLI_3306a were created as described earlier [68]. In brief, the upstream region of SCO2963 ranging from −1326 to +43 and the downstream region of SCO2962 from +2190 to +3610 were amplified by PCR from the *S. coelicolor* as described in [69] and cloned into the unstable shuttle vector pWHM3 [70]. An engineered *Xba*I site was used for insertion of the apramycin resistance cassette *aacC4* flanked by *loxP* sites between the flanking regions. The presence of the *loxP* recognition sites allows the efficient removal of the apramycin resistance cassette following the introduction of a plasmid pUWLcre expressing the Cre recombinase [71,72].

A Redirect mutant for *matB* (SCO2962) in *S. coelicolor* was made using primers matB_FW_REDIRECT and matB_REV_REDIRECT (Additional file 1: Table S3) as described previously [37].

A construct for the complementation of the mutants of SLI_3306a in *S. lividans* was designed. For this, the region of SLI_3306a from −500 to +1485 (relative to the start of *matA*) were amplified by PCR from *S. lividans* genomic DNA. This fragment was ligated into pSET152, an *E. coli*-*Streptomyces* shuttle vector which integrates in the genome at the ΦC31 attachment site [73].

#### Creating mutants using knock-out cosmids

Knock-out cosmids [35] were obtained from the collection of Paul Dyson (Swansea, UK). For details see Additional file 1: Table S2. The cosmids were introduced into *S. coelicolor* and *S. lividans* by conjugative transfer and selected for apramycin resistance, while nalidixic acid was used to prevent growth of the *E. coli* donor strain. After several rounds of non-selective growth, colonies were selected for loss of kanamycin resistance, which is the marker for the cosmid sequences. Ex-conjugants with the expected phenotype (Apra^R^/Kana^S^) were checked by PCR for absence of the wild-type gene.

### Microscopy

The mycelial morphology of *Streptomyces* in liquid-grown cultures was monitored using a Zeiss Standard 25 phase-contrast microscope and colony morphology of surface-grown cultures were imaged using a Zeiss Lumar V12 stereomicroscope as described [74].

### Tyrosinase activity assay

The specific activity of the tyrosinase enzyme produced by the *S. lividans* transformants with pIJ703 [38] was determined as described earlier [66] by measuring over time the conversion of l-3,4-dihydroxyphenylalanine spectrophotometrically at a wavelength of 475 nm.

### Whole genome sequencing and SNP analysis

Genomic DNA was isolated from liquid-grown cultures as described previously [75]. Paired-end sequencing using an ILLumina HiSEQ 2000 sequencer and mapping of the individual reads against the *Streptomyces lividans* 66 reference genome was performed at Baseclear BV (Leiden). Genome annotation was performed as described [76]. Variants were detected using the CLC Genomics Workbench version 6.5. The initial list of variants was filtered using the Phred score and the number of false positive was reduced by setting the minimum variant frequency to 70% and the minimum reads that should cover a position was set to 10. All variants were curated manually and verified by PCR analysis and routine DNA sequencing.

## Additional file

Additional file 1: Figure S1. Phenotypes of disruption mutants of *S. coelicolor* in submerged cultures. **Figure S2.** Identification of suppressor mutations in *S. lividans* PM01 and PM02. **Table S1.** Bacterial strains. **Table S2.** Transposon-mediated gene-replacement cosmids. **Table S3.** Plasmids and constructs. **Table S4.** Oligonucleotides.

## Abbreviations

aa: Amino acid
bp: Base pair
nt: Nucleotide
PCR: Polymerase chain reaction
SNP: Single nucleotide polymorphism
