# Supplementary material for: A novel locus for mycelial aggregation forms a gateway to improved Streptomyces cell factories
Source: Microb Cell Fact. 2015 Apr 1;14:44. doi: 10.1186/s12934-015-0224-6 (PMC4391728; doi:10.1186/s12934-015-0224-6)
Supplement: Additional file 1: Figure S1. — Phenotypes of disruption mutants of S. coelicolor in submerged cultures. Figure S2. Identification of suppressor mutations in S. lividans PM01 and PM02. Table S1. Bacterial strains. Table S2. Transposon-mediated gene-replacement cosmids. Table S3. Plasmids and constructs. Table S4. Oligonucleotides. [file 12934_2015_224_MOESM1_ESM.pdf]

ADDITIONAL FILE 1: Supplemental Information

belonging to the manuscript

A novel locus for mycelial aggregation forms a gateway to improved *Streptomyces* cell factories

by

Dino van Dissel[1], Dennis Claessen[1], Martin Roth[2], and Gilles P. van Wezel[1, *]

[1]Molecular Biotechnology, Institute of Biology, Leiden University, PO Box 9505, 2300RA, Leiden, The Netherlands;

[2] Bio Pilot Plant, Leibniz Institute for Natural Product Research and Infection Biology, Hans Knöll Institute, Adolf-Reichwein-Str. 23, 07745 Jena, Germany

[#] To whom correspondence should be addressed. Tel: +3171527430; email: g.wezel@biology.leidenuniv.nl

Running title: Morphological engineering of *Streptomyces*

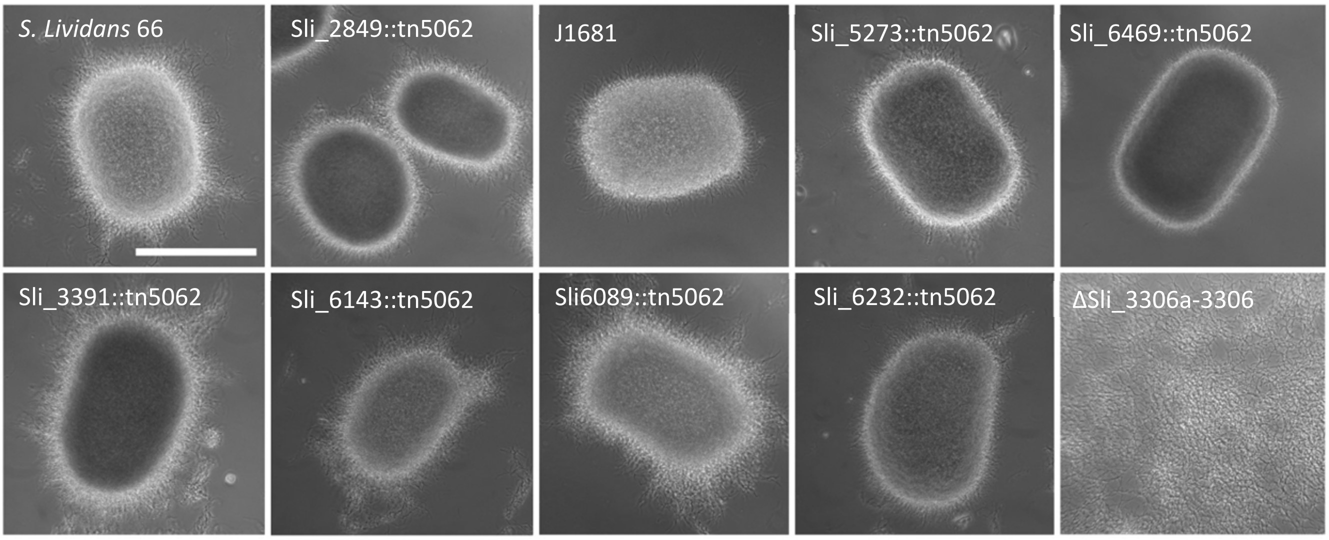

Figure S1. Phenotypes of disruption mutants of *S. lividans* in submerged cultures. SLI_2849, Sli_5273, Sli_6469, Sli_3391, Sli_ 6143, Sli_6089 and Sli_6232 were disrupted by a transposon insertion. The region of Sli_3306a-Sli_3306 was removed by homologous recombination replacement. J1681 (*S. coelicolor* Δ*bldA*) was published previously [1]. Cultures were grown in baffled shake flasks in TSBS for 48 h. Scale bar, 200 μm.

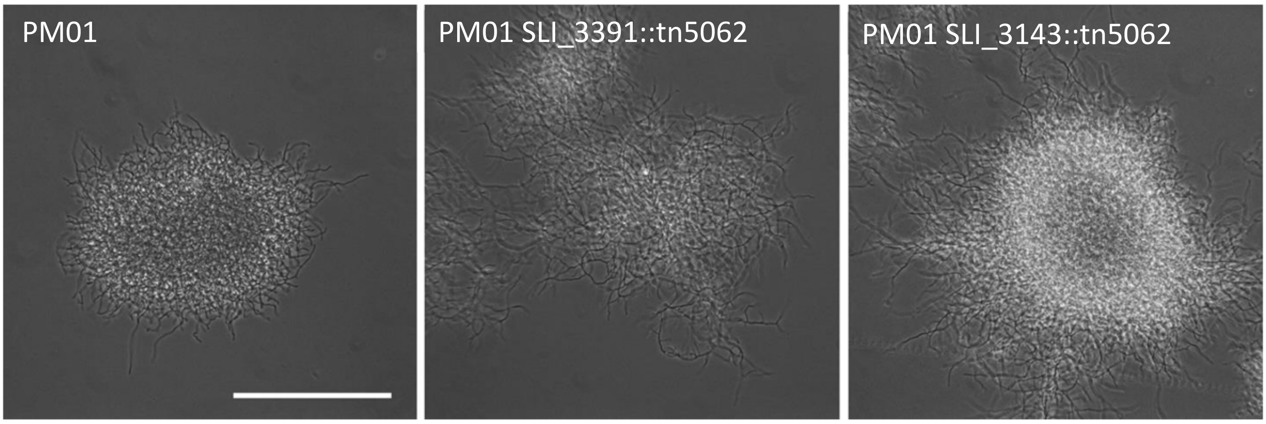

Figure S2: Identification of suppressor mutations in *S. lividans* PM01 and PM02. SLI_3391 and SLI_6143 were identified by SNP analysis as the major changes during evolution of PM02 from PM01. Note that mutation of SLI_3391 enhanced dispersed growth of PM01, giving a phenotype similar to that observed for PM02. Scale bar, 100 μm.

Table S1. Bacterial strains.

Strain	Description and genotype	Reference
Streptomyces lividans 66 (1326)	SLP2+ SLP3+	[2]
PM01	Evolved from *S.lividans* 66	[3]
PM02	Evolved from PM01	[4]
J1681	J1501 Δ*bldA*	[1]
GAD01	*S. lividans* 66 Δ*SLI_3306a::aacC4* Apr^R	This study
GAD02	*S. lividans* 66 Δ*SLI_3306a*^{clean}	This study
GAD03	*S. coelicolor* M145 Δ*SCO2962*::*aacC*4 Apr^R	This study
GAD04	*S. lividans* 66 Δ*SLI_3306a-Sli_3306*::*aacC*4 Apr^R	This study
GAD05	*S. lividans* 66 Δ*SLI_3306a-SLI3306*^{clean}	This study

IFD, in-frame deletion mutant;
Apr^R apramycin resistant.

Table S2. Transposon-mediated gene-replacement cosmids. Cosmid nomenclature refers to the *Streptomyces coelicolor* genome database (strepdb.streptomyces.org.uk). The genomic location of the insertion of the apramycin cassette is given for the *S. coelicolor* genome.

Cosmid name	*target gene*	*Cosmids location in genome*	*Start gene*	*position relative to start*
SCI7.2.C04	SCO1907	2043368	2044163	795
C121.1.E05	SCO2513	2709849	2709485	364
E34.2.E04	SCO3043	3331409	3331178	231
2SCK36.1.F01	SCO4998	5437068	5437222	154
SC5B8.1.F05	SCO5821	6369817	6369367	450
SC2E9.1.F02	SCO5871	6426513	6426319	194
7H1.2.H01	SCO5952	6521053	6520547	506
SC9B1.2.C03	SCO6076	6670727	6670057	670

Table S3. Plasmids and constructs.

Plasmid or construct	Description	Reference
pWHM3	Cloning vector, *colE1* replicon, *pSG5* replicon, $Thio^R$, Amp^R	[5]
pSET152	Complementation vector, o*riT* RK2, pUC18 replicon, $Apra^R$	[6]
pUWLcre	pUWLoriT derivative with *creA* gene under ermE* promoter, $Thio^R$	[7]
pMAT1	pWHM3 containing flanking regions of *S. coelicolor* SCO2963 and SCO2962 with a *aac(3)IV-loxP* XbaI inserted between them in pWHM3 EcoRI-HindIII	this work
pMAT2	pWHM3 containing flanking regions of *S. coelicolor* SCO2963 with a *aac(3)IV-loxP* XbaI inserted between them in pWHM3 EcoRI-HindIII	this work
pMAT3	Cosmid StE59 derivative in which the *matB* coding sequence was replaced by the *aac(3)IV* resistance cassette	this work
pMAT4	pSET152 containing *SCO2963* with the 500bp upstream (promoter) region	this work

Table S4. Oligonucleotides.

Name	Primer Sequence ^
matB_+2190	AGTCTCTAGAAGGCCGGTCGGATGACCACC
matB_+3610	AGTCAAGCTTCCCTGTTCACTCCCGCAACCG
matA_-1326	AGTCGAATTCCAGCCGGGCGGTGAGATTCC
matA_+43	ACTGTCTAGACGAGCACTCGTCGGCCGAAC
matA_2809	AGTCAAGCTTAGACGGTGTCGCCGTCCATC
matA_+1466	ACTGTCTAGACCCGGAGAACACCCTCTGATGG
matA_-54	TTCTTTGCCTGAGCACGGTGTGATAC
matB_+1528	TGGTACAGGACCACCCGGAAGAG
pmatA_-537	AGCTGAATTCGGCGCGTTACGAGAGCGGACTGAC
matA_1485	GATCTCTAGATCAGAGGGTGTTCTCCGGGGACAG
matB_FW_REDIRECT	CCGGGGTCGGCCGCGTCGGGTCGTACGGCGGGGTGGTCATGTAGGCTGGAGCTGCTTC
matB_REV_REDIRECT	CCCCTCCCTCCCCCTGTCCCCGGAGAACACCCTCTGATGATTCCGGGGATCCGTCGACC

^Restriction sites are underlined. TCTAGA, XbaI; AAGCTT, HindIII; GAATTC, EcoRI.

References

1. Leskiw BK, Bibb MJ, Chater KF. **The use of a rare codon specifically during development?** *Mol Microbiol* 1991, **5**:2861-7.

2. Kieser T, Bibb MJ, Buttner MJ, Chater KF, Hopwood DA. **Practical *Streptomyce*s genetics**. 2000.

3. Roth M, Noack D, Geuther R. **Maintenance of the recombinant plasmid pIJ2 in chemostat cultures of *Streptomyces lividans* 66 (pIJ2)**. *J Basic Microbiol* 1985, **25**:265-71

4. Roth M, Hoffmeier C, Geuther R, Muth G, Wohlleben W. **Segregational stability of pSG5-derived vector plasmids in continuous cultures of *Streptomyces lividans* 66**. *Biotechnol lett* 1994, **16**:1225-30.

5. Vara J, Lewandowska-Skarbek M, Wang YG, Donadio S, Hutchinson CR. **Cloning of genes governing the deoxysugar portion of the erythromycin biosynthesis pathway in *Saccharopolyspora erythraea* (*Streptomyces erythreus*)**. *J Bacteriol* 1989, **171**:5872-81.

6. Bierman M, Logan R, O'Brien K, Seno ET, Rao RN, Schoner BE. **Plasmid cloning vectors for the conjugal transfer of DNA from *Escherichia coli* to *Streptomyces* spp**. *Gene* 1992, **116**:43-9.

7. Fedoryshyn M, Welle E, Bechthold A, Luzhetskyy A. **Functional expression of the Cre recombinase in actinomycetes**. *Appl Microbiol Biotechnol* 2008, **78**:1065-70.
